# ADP-ribosylation of RNA and DNA: from *in vitro* characterization to *in vivo* function

**DOI:** 10.1093/nar/gkab136

**Published:** 2021-03-08

**Authors:** Lisa Weixler, Katja Schäringer, Jeffrey Momoh, Bernhard Lüscher, Karla L H Feijs, Roko Žaja

**Affiliations:** Institute of Biochemistry and Molecular Biology, RWTH Aachen University, Pauwelsstrasse 30, Aachen, Germany; Institute of Biochemistry and Molecular Biology, RWTH Aachen University, Pauwelsstrasse 30, Aachen, Germany; Institute of Biochemistry and Molecular Biology, RWTH Aachen University, Pauwelsstrasse 30, Aachen, Germany; Institute of Biochemistry and Molecular Biology, RWTH Aachen University, Pauwelsstrasse 30, Aachen, Germany; Institute of Biochemistry and Molecular Biology, RWTH Aachen University, Pauwelsstrasse 30, Aachen, Germany; Institute of Biochemistry and Molecular Biology, RWTH Aachen University, Pauwelsstrasse 30, Aachen, Germany

## Abstract

The functionality of DNA, RNA and proteins is altered dynamically in response to physiological and pathological cues, partly achieved by their modification. While the modification of proteins with ADP-ribose has been well studied, nucleic acids were only recently identified as substrates for ADP-ribosylation by mammalian enzymes. RNA and DNA can be ADP-ribosylated by specific ADP-ribosyltransferases such as PARP1–3, PARP10 and tRNA 2′-phosphotransferase (TRPT1). Evidence suggests that these enzymes display different preferences towards different oligonucleotides. These reactions are reversed by ADP-ribosylhydrolases of the macrodomain and ARH families, such as MACROD1, TARG1, PARG, ARH1 and ARH3. Most findings derive from *in vitro* experiments using recombinant components, leaving the relevance of this modification in cells unclear. In this Survey and Summary, we provide an overview of the enzymes that ADP-ribosylate nucleic acids, the reversing hydrolases, and the substrates’ requirements. Drawing on data available for other organisms, such as pierisin1 from cabbage butterflies and the bacterial toxin–antitoxin system DarT–DarG, we discuss possible functions for nucleic acid ADP-ribosylation in mammals. Hypothesized roles for nucleic acid ADP-ribosylation include functions in DNA damage repair, in antiviral immunity or as non-conventional RNA cap. Lastly, we assess various methods potentially suitable for future studies of nucleic acid ADP-ribosylation.

## INTRODUCTION

The posttranslational modification of proteins with polymers of ADP-ribose (PARylation) was first identified in the sixties (1,2). In the following decades, an essential role of poly(ADP-ribose) (PAR) in the DNA damage response was described, as summarized elsewhere (3). The enzymes generating PAR, most notably PARP1, have successfully been employed in the treatment of certain types of cancer, such as breast and ovarian cancers (4). In parallel, bacterial toxins were identified that modify specific substrates in host cells with monomers of ADP-ribose (MARylation) as part of their toxic principle (5,6). The field of ADP-ribosylation has expanded in recent years; it has become clear that only a minority of the ADP-ribosyltransferases (ARTs) are capable to form PAR-chains, while the majority of enzymes are mono(ADP-ribosyl)transferases that attach a single ADP-ribose (ADPr) to their targets (2,7). Different ARTs modify different amino acid acceptors, such as glutamate or serine. This ADP-ribosylation of proteins can for example directly change protein activity or influence interactions with other macromolecules (8). ADP-ribosylation of proteins is for example intimately involved in DNA damage repair, signalling and RNA regulatory processes, as reviewed elsewhere (2,9). Recently, several groups reported ADP-ribosylation of oligonucleotides, both RNA and DNA, by mammalian ARTs that were thought to modify exclusively proteins. In this Survey and Summary, we first provide an overview of the transferases performing this newly identified nucleic acid modification, the hydrolases reversing it and the characteristics that define suitable oligonucleotide substrates for the various ARTs. We extrapolate from data available for nucleic acid ADP-ribosylation in different species to speculate about potential functions in mammalian cells and summarize the possibilities for detecting ADP-ribosylation of nucleic acids in cells. Lastly, we provide an outlook for future work, aimed at deciphering the physiological role of nucleic acid ADP-ribosylation.

## ADP-RIBOSYLTRANSFERASES

**Figure 1.**
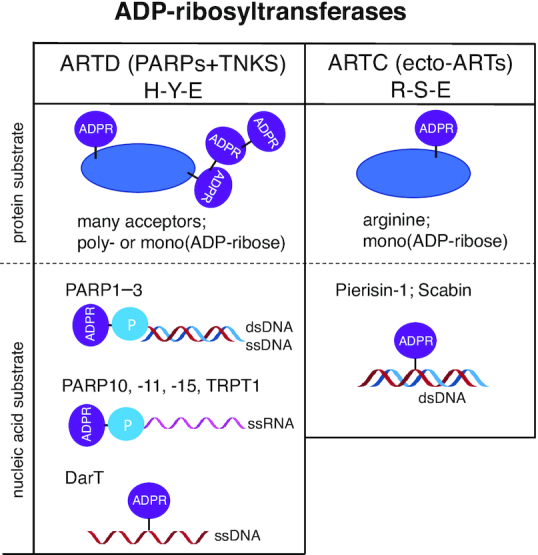
ADP-ribosyltransferases and their substrates. The ADP-ribosyltransferases (ARTs) can be subdivided in two subclasses based on key amino acids present in their catalytic domain: either H–Y–E or a derivate thereof for the ARTDs (including mammalian PARPs and the two tankyrases (TNKSs)), or a motif based on R–S–E for the ARTCs (including mammalian ecto-ARTs). The PARP family is most divergent and different members modify different amino acids. Some PARPs generate poly(ADP-ribose), whereas others are limited to mono(ADP-ribosyl)ation. The ecto-ARTs are more restricted and modify arginine with ADP-ribose. Several bacterial toxins of the ARTC subfamily with an R-S-E motif were shown to modify DNA internally. ARTDs show a much broader spectrum of nucleic acid substrates but appear to be dependent on a phosphate for modification, with the exception of DarT, as summarized in more detail in Table 1. Examples of relevant enzymes and substrates are shown; for PARP10, PARP11 and PARP15 only catalytic domains have shown activity towards nucleic acids.

ARTs are defined by their catalytic ART domain, a protein fold that enables binding of NAD^+^ and transfer of ADP-ribose from NAD^+^ onto a substrate under release of nicotinamide. While the catalytic domains of individual ARTs are generally poorly conserved at the sequence level, the overall structure is shared among the variety of ARTs (5). However, two distinct motifs, each comprised of three highly conserved amino acids situated within the NAD^+^ binding sites, are used to categorize the ART superfamily in bacteria as well as in their eukaryotic descendants: ARTs with a histidine–tyrosine–glutamate triad (H–Y–E motif and variants thereof) are related to the diphtheria toxin from *Corynebacterium diphtheria* and as such are referred to as ADP-ribosyltransferase diphtheria-toxin like (ARTDs), whereas the ARTs with a arginine–serine–glutamate sequence (R–S–E motif and variants thereof), first described for cholera toxin of *Vibrio cholera*, are named ADP-ribosyltransferase cholera-toxin like (ARTCs) (Figure 1) (10–12).

The 22 known human ARTs are thus categorized into two subclasses according to their NAD^+^ binding mode. The 17 intracellular ADP-ribosyltransferases known as PARPs, as well as tRNA 2′-phosphotransferase (TRPT1), belong to the ARTD subclass, while the ARTC subclass of ARTs is comprised of four ecto-ARTs (5,13). Despite divergent binding mechanisms, ARTCs and ARTDs share considerable similarities in the configuration of NAD^+^ within their binding pockets. The histidine in the H–Y–E motif of ARTDs binds the NAD^+^ at the 2′-hydroxyl of the adenosine ribose and the amine of the nicotinamide via hydrogen bonds. Simultaneously, the tyrosine interacts with the nicotinamide ribose via π-stacking and the glutamate's carboxyl stabilizes the resulting ADPr-furanose intermediate by interacting with the 2″-hydroxyl of the nicotinamide ribose. Within the bacterial R–S–E triad, the glutamate fulfils the stabilizing function and the arginine interacts with the diphosphate via electrostatic interaction, whereas the serine builds hydrogen bonds with the nicotinamide ribose (5,14,15). Apart from the catalytic triad, two loop-structures characterize the ART domain and possess important functional roles. This is especially well documented for bacterial ARTs: the acceptor loop is involved in target recognition and selectivity, while the donor loop (exclusive for H–Y–E motif ARTDs) participates in substrate specificity and in catalysis and interaction with the ADP-ribose of NAD^+^ (15,16). Even though both loop structures are evolutionary highly conserved, their primary structure and length vary greatly among the ARTDs.

The ability of ARTs to either catalyse mono-ADP-ribosylation (MARylation) or poly-ADP-ribosylation (PARylation) of their protein substrates is contingent on the catalytic triad and the presence of certain cofactors as well as additional conserved structural features. Furthermore, ARTs exhibit wide substrate specificities in regard to the acceptor amino acids (7,17,18). PARP-mediated ADP-ribosylation, for example, was first considered as a protein modification occurring on glutamate (17). Recently serine was identified as the preferred substrate for PARP1 when HPF1 is present as co-factor (17,19). Additionally, cysteine, histidine, threonine and tyrosine residues were demonstrated as sites of ADP-ribosylation, although the respective transferases and *in vivo* occurrence remain largely unknown (18,20–22). The role of proteins as acceptor molecules is comprehensively discussed in several reviews (2,9,17,23,24) and will not be further addressed here.

In addition to proteins as substrate for ADP-ribosylation, it became clear that some ARTs are capable of modifying oligonucleotides. The first DNA-targeting ART, pierisin, was discovered in pierid butterflies (25). To date, six pierisins (pierisin-1, -1b and -2–5) were identified that build together with ScARP and Scabin, found in *Streptomyces*, the pierisin family (26). These enzymes are members of the ARTC subfamily. They ADP-ribosylate the N2 position of guanine in different DNA substrates with varying sequence specificity. Replication of ADP-ribosylated DNA is significantly slower than replication of non-modified DNA and appears to be perceived as DNA damage, which is repaired by nucleotide excision repair (NER) (27). Subsequent studies have identified the same ADP-ribosylation activity in a variety of shellfish species (28). These enzymes are relatively well studied and multiple roles for DNA modifying ARTCs have been proposed. The pierisins are considered part of the defence system of cabbage butterflies against pathogens, while CARP-1 in shellfish has been proposed to be part of the innate immune response to DNA viruses and/or regulation of NAD^+^ concentration (29). These studies provide initial evidence for ADP-ribosylation contributing to host-pathogen conflicts.

Nucleic acids have only recently been identified as substrates for mammalian ARTD-mediated ADP-ribosylation and their role as potential regulator of DNA and/or RNA functions is poorly understood. Here, we will provide an overview of the ARTs and hydrolases that modify and de-modify, respectively, different DNA and RNA substrates as well as hypothesize about potential functions of this novel type of DNA/RNA modification.

### Evolutionary origins: bacterial DNA ADP-ribosylation

Toxin-antitoxin (TA) systems regulate several biological processes within bacteria, including metabolism and stress response, thereby promoting the persistence of cell populations (30). In *Thermus aquaticus*, the expression of DarT results in a bacteriostatic effect if the antitoxin darG is absent (31). The induction of DarT signals DNA damage to the repair machinery and triggers the SOS response (32). The toxin DarT was characterized as an ARTD-like enzyme capable of DNA ADP-ribosylation (31). To identify potential substrates for DarT, bacterial protein extracts, total bacterial RNA or denatured bacterial DNA were incubated with DarT and NAD^+^. Efficient incorporation of NAD^+^ was observed only when denatured DNA was used as a substrate. Consecutive experiments demonstrated that DarT ADP-ribosylates specifically ssDNA in a sequence specific manner: ssDNA oligos with a minimum length of eight bases were successfully modified at the second thymidine of a TNTC motif *in vitro* (31). As replacement of thymidine by uridine or deoxyuridine prevents ADP-ribosylation, a strict DNA specificity is presumed. This exclusive ADP-ribosylation of one specific base is counteracted by the antitoxin DarG. The observed DNA ADP-ribosylation affects replication, which might be the cause of the observed bacteriostatic effect (31). The existence of comparable TA systems was also shown in *Mycobacterium tuberculosis* and the enteropathogenic *Escherichia coli* (EPEC), where DarT also downregulates DNA replication and specifically modifies ssDNA, which is reversed by DarG (33,34). However, this toxin modifies the sequences TTT or TCT in EPEC that differ from the target sequence of DarT in *T. aquaticus*. Expression of RecF, which recognizes ssDNA breaks (35), is crucial for cell survival after DarT induction. Therefore, it was suggested that cells perceive DNA ADP-ribosylation as DNA damage, which is antagonized by NER, similar to the modification introduced by pierisin-1 (27).

These studies highlight that ARTD enzymes can modify DNA in an unexpected, sequence specific manner. This adds DNA, in addition to proteins, to the list of substrates of ADP-ribosylation. Furthermore, these examples hint at the possibility that also mammalian ARTs might be able to ADP-ribosylate nucleic acids.

### Mammalian DNA ADP-ribosylation

PARP1, the first discovered and best studied PARP, is well-known for its role in DNA damage repair, where it is responsible for the recruitment of numerous DNA repair factors upon activation and PAR chain synthesis (36). PARP1 is specifically linked to base excision repair (BER) and NER where it produces PAR chains as scaffold for the DNA repair machinery. Furthermore, it is involved in homologous recombination and other cellular processes like the modulation of the chromatin structure and gene expression (23,37,38).

The three DNA-associated PARPs (PARP1–3) differ in their structure but commonly harbour a tryptophan–glycine–arginine (WGR) domain, essential for DNA binding, and a C-terminal (CTD) domain with coincident three-dimensional structure (39–41). Furthermore, the enzymatic activities of PARP1–3 are regulated by an inducible conformational change within their catalytic domains, leading to a local stabilization or destabilization within the NAD^+^ binding sites (42,43). Upon activation these PARPs are able to modify themselves and other proteins (44,45). PARP1 has a modular architecture that consists of six independent domains with diverse functions that are connected by flexible linkers (46,47). Activation of PARP1 is enabled by this complex modular architecture through an allosteric mechanism that induces a conformational change of the whole multi-domain folding upon interaction with damaged DNA. Three flexible N-terminal zinc-fingers (ZF1–3) recognize the exposed bases of damaged DNA. PARP1 bends the DNA duplex at the strand break via cooperative action of ZF1 and ZF2, each binding to one of the exposed DNA breakage sites. ZF1, binding to the 5′-end, is required for the activation at double strand breaks (DSB) whereas ZF2, binding to the 3′-end, is responsible for the recognition of single strand breaks (SSB) (48,49). In a model using an SSB DNA mimic, bending of the DNA by ZF1 and ZF2 enables the coordination of ZF3 that interacts with ZF2 and the DNA. The induced allosteric interactions result in the local unfolding of an auto-inhibitory helical subdomain within the ART module that enables catalytic activity of PARP1 (42,45,48–52). A more detailed discussion about the PARP1 DNA binding modes and their functions is given elsewhere (53).

This interaction with damaged DNA, particularly SSBs and DSBs, brings PARP1 into close vicinity of the 5′- and 3′-ends of DNA that constitute potential nucleophiles able to promote ADP-ribosylation. Indeed, PARylation activity of PARP1 and PARP2 on phosphorylated termini of DNA oligonucleotide duplexes and ssDNA oligomers was demonstrated *in vitro*. Depending on the structure and localization of DNA strand breaks, PARP1 ADP-ribosylates 3′- and 5′-terminal phosphates of dsDNA and ssDNA as well as exposed 5′-phosphates of gapped dsDNA, although with very weak efficiency (54–56) (Table 1). PARP2 seems unable to modify termini of intact DNA duplexes, but prefers 5′-phosphorylated blunt ends of recessed, nicked and gapped dsDNA (57). ssDNA provokes weak ADP-ribosylation activity of PARP2 on both 3′- and 5′-terminal phosphates (56). Beside these PARylating PARPs, PARP3, which MARylates proteins, is also capable to modify DNA substrates. The 5′- and 3′-phosphorylated groups of dsDNA blunt ends were confirmed as ADPr acceptor nucleophiles for PARP3, whereby the 5′-end presents the preferred target (55,57). Additionally, exposed 5′-phosphorylated ends of recessed dsDNA (55,57,58) and exposed 5′-phosphates of nicked dsDNA (55,57,58) were examined as targets with contradictory results (Table 1). The DNA substrates used for these studies differ in terms of length and nucleic acid sequence. It is not known whether PARPs that are active on nucleic acids possess sequence specificity or whether complex DNA or RNA structures are required to enable binding and/or enzymatic activity. Also the insertion of chemical compounds, like cordycepin-monophosphate (an AMP mimic) and phosphoribosyl-AMP, on the acceptor sites of the nucleic acid substrates could influence the enzymatic activities and specificities. Additionally, the enzymes used in the published studies differ in their procedure of protein expression and purification. The latter is particularly relevant as proteins synthesized in mammalian or insect cells differ greatly in their level of posttranslational modifications compared to recombinant proteins produced in bacterial expression systems.

**Table 1.** Nucleic acid substrate modification by PARP1–3. The different potential substrates are schematically displayed. Illustrated are the different oligonucleotides that are either single or double stranded, with or without nicks, gaps or single strand overhangs. Moreover, the positions of phosphate groups are indicated. Exact information on sequence and oligonucleotide length is provided in the studies cited.

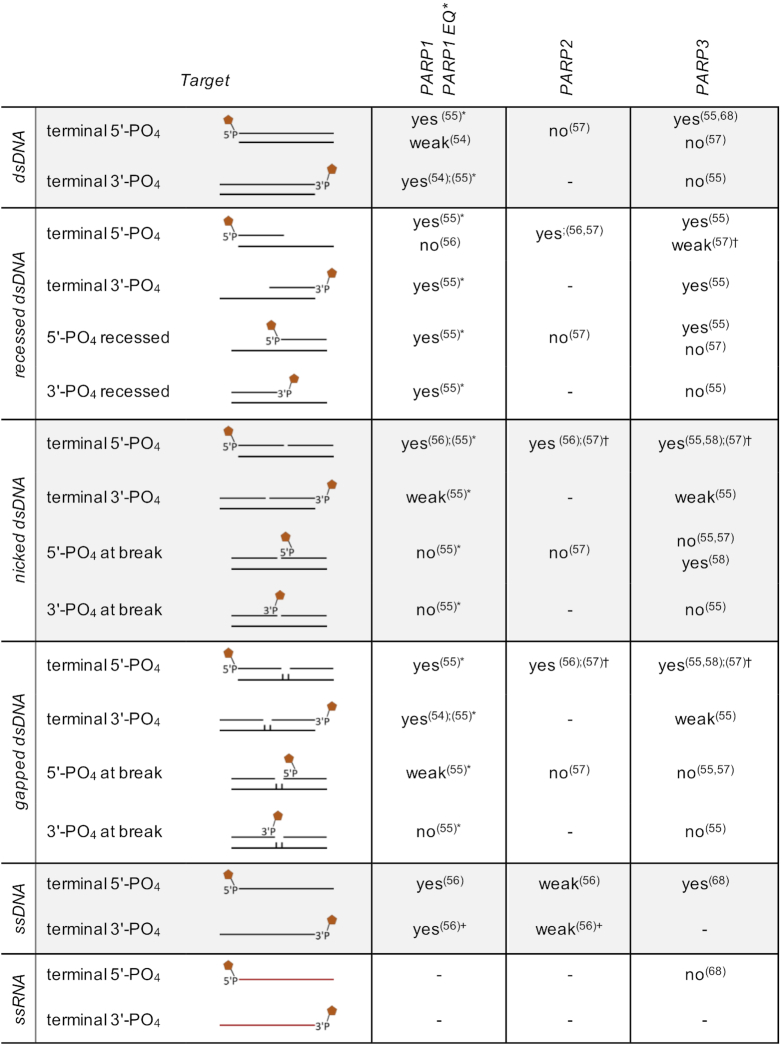

* PARP1 E998Q was used, a mutant that MARylates substrates but is unable to produce PAR; ^†^ The activity depends on the location of the nick/gap (distance should not exceed ∼21 nt); + cordycepin 5′-[^32^P]monophosphate (3′-dAM^32^P) was used as ADP-ribosylation target (the 2′-hydroxyl group of the cordycepin moiety at the 3′-end should resemble that of the ADP-ribose unit in PAR).

PARP2 and PARP3 show tendencies to preferably modify DNA strand breaks over auto-modification (>5-fold for PARP2 and >50-fold for PARP3) depending on the DNA substrates and reaction conditions (57). This tendency seems to be sequence independent but contingent on the configuration and localization of the strand break within the DNA duplex, as a distance of one or two helix-turns downstream of a DNA break appears to be required for efficient modification.

Interestingly, an incorporated ADPr at the phosphorylated 5′-end of gapped oligonucleotides can serve as primer for further modification by PARP1 and PARP2. This allows the extension of the initial MARylation to PAR chains via glycosidic 1″–2′ linkages (58). The overall exceeding processivity of PARP1 (57,59) is countered by the surpassing activity of PARP2 on PARP3-mediated ADPr primed DNA substrates (58), granting speculation about protein dimerization and/or interaction among the DNA-dependent PARPs. In this context, it is noteworthy that, depending on the DNA structure, PARP2 can indeed bind in two modes. As monomer it preferentially interacts with DNA that mimics SSBs, while as dimer binding to phosphorylated dsDNA is favoured (60,61). Also the distance-dependent activation of PARP3 and PARP2 on DNA with two damage sites could be traced back to a similar dimeric-binding mode. However, also a divergent monomeric protein binding mode can be suggested: monomeric PARP2 or PARP3 modifies a single-strand break within a DNA duplex, depending on the activation by a double-strand break in proximity (57). This could be based on the recognition of one of the DNA damage sites by the WGR domain that brings the second strand-break in proximity to the catalytic domain to enable modification, while exceeded or reduced distance to the DSB could sterically hinder the interaction between the SSB and the active site. Another possible scenario could be a scaffold function of an initially bound PARP that recruits a second PARP molecule, either as homo- or heterodimeric interaction, that modifies the second DNA strand break. These findings suggest that PARP3 has strong MARylation activity towards DNA, while auto-modification appears to be very low. Interestingly, the ADP-ribose mark deposited on DNA by PARP3 serves as primer for PARylation by PARP1 and PARP2. Together, these findings allow to postulate that PARP3′s primary function could be the modification of DNA in order to directly mark DNA lesions. This would be a novel function of ADP-ribose at DNA breaks, in addition to the commonly accepted model of ADP-ribosylated PARPs serving as scaffolds.

DNA repair processes are often performed by ATP-dependent polynucleotide ligases. During this reaction the ligase is activated by ATP, resulting in a ligase-AMP intermediate, that transfers AMP to the exposed 5′-phosphate of the DNA damage site. This marks and activates the DNA breakage and enables the attack of the 3′-hydroxyl on the 5′-phosphate to from a phosphodiester bond under release of AMP (62). As PARP3 can MARylate 5′-phosphorylated SSBs of DNA, it was tested whether the presence of ADPr can induce ligation of DNA strand breaks. Indeed, ADPr seems to enable ligation of SSBs by ATP-dependent DNA ligases in the absence of ATP (58). Co-immunoprecipitation (co-IP) studies of PARP3 identified various proteins that are involved in the non-homologous end joining (NHEJ) DNA repair process (63,64). The activity of PARP3 on damaged DNA together with the observations of the co-IP studies suggest an involvement of DNA MARylation by PARP3 in DNA repair pathways (58).

NHEJ mediates the direct re-ligation of DNA lesions without the requirement of a homologous template (65). In this context, the modification of DNA SSBs by PARP3 could have a protective effect on DNA lesions by shielding the exposed ends until repair protein complexes are recruited. As PARP1 and PARP2 can extend the initial ADPr added by PARP3, this hypothesis is supported by the finding that PARylated DNA breakage sites are protected from degradation by endonucleases (56). Evidence of a strong activation of PARP2 and PARP3 in response to 5′-phosphorylated DNA breaks further substantiates a potential involvement of these proteins in DNA repair mechanisms (43,66). The reduction of chromosomal rearrangements in cells depleted of PARP3 also supports its participation in the NHEJ pathway (67).

Collectively, these studies demonstrate that PARP1–3 are capable of modifying a range of DNA substrates with either PAR or MAR *in vitro* (Table 1). However, despite the biochemical characterization of ADP-ribosylation at DNA breakage sites, the function of this novel DNA modification in cells remains to be unravelled.

### ADP-ribosylation of RNA by PARPs

RNA, in addition to DNA, has been demonstrated to serve as substrate for ADP-ribosylation catalysed by mammalian PARPs. The activities of several PARPs and hydrolases were tested on a variety of RNA substrates *in vitro* (68). The activity of PARP10 on phosphorylated RNA termini has been demonstrated with a preference of 5′-phosphorylated over 3′-phosphorylated ssRNA substrates (68). However, PARP10 is not able to modify ssDNA, which is in contrast to PARP3 that is active on ssDNA but unable to MARylate RNA (57,68). Beside PARP10, the catalytic domains of PARP11 and PARP15 are also capable of modifying phosphorylated ssRNA (Table 2). Full-length PARP3 and PARP16 as well as the catalytic domains of PARP4, PARP6, PARP12, PARP13 and PARP14 were tested, however, these do not show activity on 5′-phosphorylated RNA (68). Because the catalytic activity of the enzymes used in this study was not verified on protein substrates, and thus a priori functionality was not documented, the results should be interpreted with caution.

**Table 2.** Overview of the oligonucleotides modified by the catalytic domains of PARP10, PARP11 and PARP15 as well as TRPT1. The different potential substrates are schematically displayed. Exact information on sequence and oligonucleotide length is provided in the studies cited.

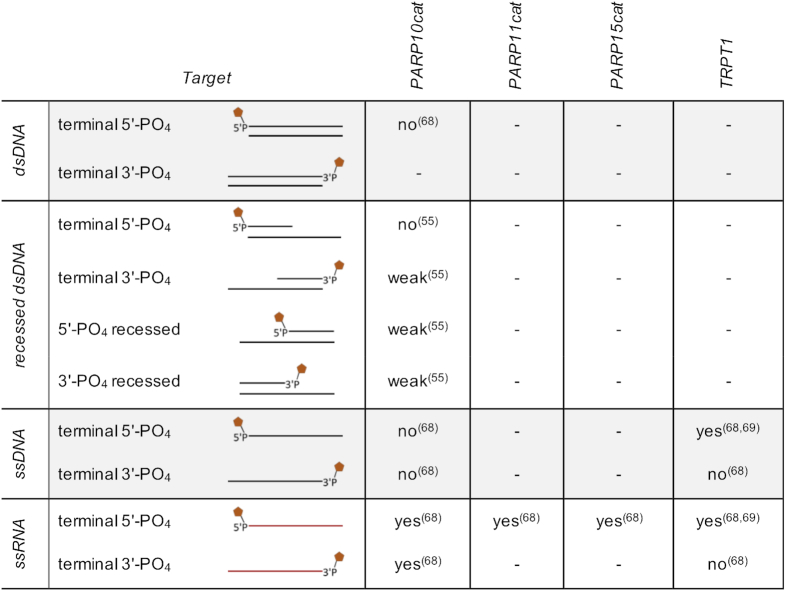

Interestingly, a truncated version of PARP10, which only contains the catalytic domain, modifies RNA more efficiently than the full-length protein. It was speculated that this is caused by an auto-inhibitory mode involving an interaction of N-terminal sequences with the catalytic domain, similar to what has been described for PARP1 (68). However, this hypothesis has not been further substantiated. PARP10 lacks an auto-inhibitory, helical structure in its catalytic domain, which argues against an allosteric activation mechanism similar to PARP1–3, as discussed above. Instead, the full-length protein with its RNA recognition motif (RRM) together with the glycine-rich region may be regulated by binding to specific RNAs. PARP10 also contains ubiquitin interaction motifs (UIM), which allow interaction with poly-ubiquitin chains (70), and a PIP box that enables the interaction with the DNA clamp PCNA, a processivity factor that is essential for cell proliferation (71,72). The binding of HPF1 to PARP1 and PARP2 demonstrates the ability of interaction partners to alter substrate specificity (61,73), which could also be the underlying concept of the regulation of PARP10 in terms of nucleic acid modification.

Apart from the observation that the catalytic domains of PARP11 and PARP15 can transfer ADP-ribose onto RNA *in vitro*, no further studies have been published regarding these enzymes. This leaves the question unanswered whether the full-length proteins possess comparable activity towards RNA (68). Moreover, it will be interesting to determine whether these enzymes are capable to modify RNA in cells. The various PARPs, which exhibit activity towards RNA substrates, localize to different intracellular compartments where they could fulfil comparable biochemical reactions. For example, PARP10 is localized to cytoplasmic structures containing p62 and ubiquitin and can shuttle into the nucleus and nucleolus depending on its phosphorylation status (74,75), whereas PARP11 is located in the nuclear envelope (76) and PARP15 in stress granules (77). Different subcellular localizations provide a tenable explanation for a number of PARPs that display similar enzymatic activities, complementing each other in different compartments and in different cellular processes. The proteins might be differently regulated in response to signalling cascades or by interacting with distinct, localization-specific co-factors. At present, our knowledge is rather poor about how different ARTs are controlled regarding activity and substrate specificity.

Similar to DNA ADP-ribosylation, little is known about the role of RNA ADP-ribosylation in cells. The canonical m7Gppp cap protects nascent RNA from premature degradation by 5′→3′ exonucleases, promotes the translocation of mature mRNA into the cytosol, and is essential for initiation of translation (78). In addition, the m7Gppp cap also serves as a scaffold to recruit protein factors involved in RNA processing. On the contrary, the recently discovered non-canonical NAD^+^ capping by RNA polymerase II blocks efficient translation and actively targets the RNA for degradation (79,80). In light of these observations and the structural similarities of NAD^+^ and ADPr, it would be interesting to explore whether ADPr has a similar effect on RNA in regards to mRNA stability, localization and translation.

### ADP-ribosylation of RNA by TRPT1

TRPT1 was originally described as an essential component of the fungal tRNA splicing machinery. Following the excision of introns, the resulting exons are joined together by ligases leaving a 3′–5′ phosphodiester splice junction that possesses a characteristic 2′-PO_4_, which has to be removed in order to generate mature tRNA (81). This reaction proceeds in two steps, culminating in a short lived 2′-phospho-ADP-ribosylated RNA intermediate.

TRPT1 homologs are evolutionary conserved in eukarya, bacteria and archea. Its deletion in yeast is lethal, but complementation assays with mammalian and bacterial TRPT1 homologs showed conservation of catalytic specificity in removing the tRNA 2′-PO_4_ through a transfer to NAD^+^ that generates mature 2′-OH RNA (82,83).

Bacterial, archeal and metazoan tRNA processing is different from that of fungi and does not result in a 2′-PO_4_ junction. This poses the question why enzymes that are capable of dephosphorylating 2′-PO_4_ of RNA, generating ADP-ribose 1″–2″ cyclic phosphate, exist in prokaryotes and metazoan. They might have an as of yet unidentified biochemical pathway enabling the production of 2′-phosphorylated RNA. RNA modified in this way would then act as a potential substrate for TRPT1 mediated RNA repair. In two recent studies, prokaryotic and fungal TRPT1 were characterized for their ADP-ribosylation activity (68,69). The TRPT1 homologs are able to transfer ADPr moieties to the phosphate group of 5′-monophosphorylated ssDNA and ssRNA *in vitro*. This ADP-ribosylation resulted in a phosphatase-resistant 5′-phospho-ADPr cap structure on 5′-DNA/RNA substrates. Based on the first reaction step of the tRNA 2′-phosphotransferase mechanism, a potential one-step reaction via an ADPr transferase reaction was suggested resulting in capping of DNA and RNA (69). TRPT1 was identified in humans as a phosphate-dependent mono-ART, which exclusively modifies 5′-PO_4_ of ssDNA and ssRNA substrates, independent of oligomer length (68). The unexpected modification of RNA by TRPT1 offers a potential explanation for the existence of the catalytically active TRPT1 even in taxa that lack splicing systems.

## REVERSAL OF DNA/RNA ADP-RIBOSYLATION

Two protein families are known that reverse ADP-ribosylation. The macrodomain-containing family and the ADP-ribosylhydrolase family (ARHs). Macrodomain-containing proteins are conserved through all domains of life and are defined by the presence of a macrodomain fold, named after the non-histone region of the histone MacroH2A, which is characterized by a mixed α/β fold with structural resemblance to certain nucleotide hydrolases (84–88). The mammalian ARH family contains three proteins with structurally similar catalytic domains, ARH1, ARH2, and ARH3 (89–92). The three members share similar amino acid sequences, where ARH1 and ARH2 display a higher degree of sequence homology (92). The following section addresses members of both protein families that have hydrolytic activity towards ADP-ribosylated DNA or RNA substrates.

### ADP-ribosylhydrolase family

**Figure 2.**
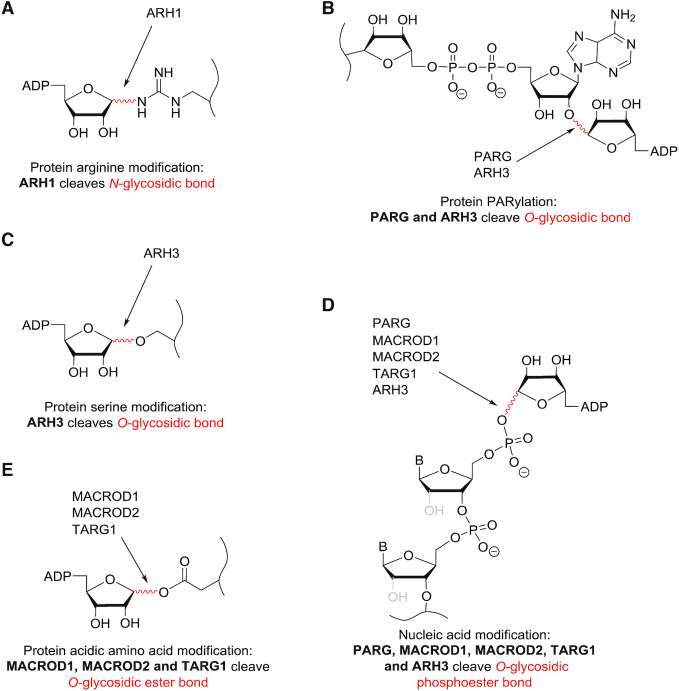
Substrate specificities of proteins that reverse ADP-ribosylation. (**A**) ARH1 cleaves the *N*-glycosidic bond in modified arginine residues. (**B**) The *O*-glycosidic bond in PAR-chains is broken by PARG and ARH3 or (**C**) the *O*-glycosidic bond present in serine-ADPr is broken by ARH3, respectively. (**D**) PARG, MACROD1, MACROD2, TARG1 and ARH3 have all been demonstrated to remove ADPr from DNA and RNA by cleaving the phosphoester type *O*-glycosidic bond (**E**) ADP-ribosylation of acidic amino acids is resolved by MACROD1, MACROD2 and TARG1.

The members of the ARH family harbour an evolutionary highly conserved catalytic domain of 290–360 residues. The first described ARH enzyme was DraG (dinitrogenase reductase activating glycohydrolase) from *Rhodospirillium rubum*, which plays an essential role in nitrogen fixation (93). Biochemical characterization has shown that DraG specifically reverses arginine-ADP-ribosylation. An enzyme with the same activity, ARH1, was later identified in animal cells (94). In addition to ARH1, ARH3 is also catalytically active, while the third mammalian ARH family member, ARH2, appears to be inactive, possibly due to the lack of an aspartate in the catalytic centre (92,95). ARH1 and ARH3 display different substrate specificities. The primary activity of ARH1 is the hydrolysis of the *N*-glycosidic bond between ADPr and arginine residues (94,96–99) (Figure 2A). Only weak activity against PAR and *O*-acetyl-ADP-ribose (*O*AADPr) has been detected. Although the function of ARH1 is not fully understood, experiments in mouse embryonic fibroblasts deficient for ARH1 showed rapid accumulation of arginine-ADPr substrates (100), correlating with abnormal cell proliferation. So far, ARH1 is the only mammalian enzyme that is able to cleave the *N*-glycosidic bond between ADPr and arginine. The finding that ARH1 is not able to remove ADP-ribose from nucleic acids is in accordance with the previously described substrate specificity of ARH1, since the linkage between ADP-ribose and DNA/RNA is *O*-glycosidic.

ARH3 was originally described as a PAR glycohydrolase (Figure 2B) (92). Besides PAR, ARH3 efficiently uses *O*AADPr as a substrate (98,101). Various inactivating mutations in the *ADPRHL2* gene have been identified in individuals with neurodegenerative disease (102–104), hinting at the importance of ARH3. It is not understood yet how loss of functional ARH3 would lead to this phenotype. Recent research shows that ARH3 possesses unique activity towards MARylated serines, which seems to be the predominant type of modification during the DNA damage response (105,106) (Figure 2C). Nuclear localization of ARH3 supports its role in DNA damage response. However, ARH3 also localizes to the cytoplasm and mitochondria where serine modification has not been detected (92,107), leaving the role of ARH3, especially in mitochondria, elusive. Although no ART has been identified in mitochondria, several sirtuins are present in this organelle. Sirtuins act as deacetylases using NAD^+^ as cofactor resulting in the formation of *O*AADPr (108). Consequently, a possible role of ARH3 could be the degradation of *O*AADPr in the recycling of mitochondrial NAD^+^. In accordance with its specificity towards *O*-glycosidic bonds in the substrates detected so far (*O*AADPr, Ser-ADPr and PAR), ARH3 cleaves ADPr from DNA and RNA, the proposed linkage being a phosphoester-type *O*-glycosidic bond (Figure 2D) (55,68).

### Macrodomain-containing hydrolases

After the degradation of PAR chains via the hydrolysis of ribose–ribose bonds was observed (109), poly(ADP-ribose)glycohydrolase (PARG) was identified as responsible enzyme (110) (Figure 2B). Structural analyses of bacterial and protozoan PARG revealed a macrodomain fold, containing a unique loop sequence within the ADPr binding site that harbours a catalytic glutamate residue (111,112). PARG is encoded by a single gene in mammals (113), but is expressed as several isoforms due to alternative splicing (114). Full-length human PARG (111 kDa) is targeted to the nucleus, while other isoforms are located in the cytoplasm (114). Knockout of *PARG* results in early embryonic lethality in mice (115). Cells derived from trophoblasts of PARG knockout embryos only survive in the presence of the broad-spectrum PARP inhibitor benzamide (16,115), suggesting that the lack of PAR turn-over and its accumulation is toxic, making the reversal of ADP-ribosylation by PARG in mammals essential. In contrast to the results in mice, the absence of a catalytic active PARG in *Drosophila melanogaster* results in high lethality. The survived flies show an accumulation of PAR in nervous tissue along with progressive neurodegeneration (116). Accumulation of PAR chains in the cytosol has also been described to be accompanied by induction of apoptosis in a process referred to as parthanatos (117,118).

Besides its activity towards protein bound as well as free PAR chains, PARG can remove ADPr and PAR units bound *via O*-glycosidic phosphoester bonds to 3′- and 5′-phosphates of dsDNA (55) (Figure 2D). Furthermore, PARG is capable to efficiently reverse MARylation of 3′- and 5′-phosphorylated ssRNA (68). The observed activities toward ADP-ribosylated DNA and RNA fit to the specificity of PARG for *O*-glycosidic linkages (Figure 2B and D).

Aside from PARG, three other human macrodomain-containing proteins possess hydrolytic activity that is selective for ADPr modified substrates: MACROD1, MACROD2 and TARG1. These enzymes were first demonstrated to hydrolyse *O*AADPr, the by-product of sirtuin mediated deacetylation (119,120). Later it was found that MACROD1, MACROD2 and TARG1 specifically remove MAR from proteins modified at acidic amino acid residues (Figure 2E) (121–124), but not PAR, making these enzymes specialized MAR hydrolases (121,125,126). Additionally, MACROD1, MACROD2 and TARG1 efficiently reverse oligonucleotide modification by hydrolytic cleavage of ADP-ribose from the 5′ or 3′ terminal phosphates of dsDNA as well as ssRNA (Figure 2D) (55,68,127).

Relatively little is known about the physiological function of these macrodomain-containing hydrolases. MACROD1 is a mitochondrial protein, highly expressed in skeletal muscle and certain breast cancers (127–130). MACROD2 is present in the nucleus and the cytoplasm (128–130). TARG1, which is the most ubiquitously expressed protein of these three, is mainly localized in the nucleus (123,129,131,132). A few studies have addressed their localization in response to certain stimuli and found that overexpressed MACROD2 is exported from the nucleus into the cytoplasm upon phosphorylation by the ataxia-telangiectasia-mutated kinase, which is activated in response to DNA damage (132). A mutation in the *OARD1* gene, which encodes the TARG1 protein, was identified in patients with a severe neurodegenerative phenotype, although it was not clarified how loss of TARG1 leads to this illness (123). TARG1 predominantly localizes to transcriptionally active nucleoli, but relocalizes to the nucleoplasm when rRNA transcription is inhibited (131). Upon DNA damage TARG1 accumulates in the nucleoplasm and at sites of damaged DNA, depending on PAR formation (123,131). Together this suggest that TARG1 is involved in the DNA-damage response and/or its nucleolar function needs to be halted in response to DNA damage. Moreover, TARG1 binds to RNA oligomers (131), possibly through positively charged surface patches, as suggested for the Chikungunya viral macrodomain (133). This activity of TARG1 might be relevant for targeting ADP-ribosylated RNA substrates. MACROD1 and TARG1 interactomes revealed a number of nucleic acid metabolism associated proteins such as helicases and nucleases (129,131). As described above, MACROD1 and TARG1 are primarily localized in mitochondria, stress granules and nucleoli, compartments of intensive nucleic acid processing. Together with the interactome results, these findings are in line with a potential role of macrodomain-containing hydrolases in nucleic acid metabolism.

It is not well understood how PARG, MACROD1, MACROD2, TARG1 and ARH3 are able to remove MAR from 5′- or 3′-phosphate groups in DNA and RNA (Figure 2D). PARG breaks down *O*-glycosidic bonds between ribose rings, but is unable to cleave the primary ADPr of amino acid acceptors (105,106,112). However, the chemical structure of the bonds between ADPr and serine and between ADPr and nucleic acids are quite similar (Figure 2). To understand why PARG is unable to remove MARylation from serine, further structural studies will be required. Structural constraints may prevent accommodation of a protein substrate within the active centre, while binding of a nucleic acid substrate is possible. Indeed, PARG is able to completely reverse DNA- and RNA-phosphate modification and even appears to be more active than MACROD2, ARH3 and TARG1 (55). As MACROD1, MACROD2 and TARG1 cleave ester-type *O*-glycosidic bonds between the protein proximal ribose of ADPr and side chains of acidic amino acids (121,123,124) or acetate groups in *O*AADPr (119,120), the hydrolysis of the *O*-glycosidic phosphoester bond present in modified nucleic acid substrates seems appropriate. ARH3 shows activity towards the *O*-glycosidic bonds in PAR chains as well as between serine and ADPr in modified proteins. Specific conformational requirements, amino acid residue, surrounding amino acid sequences or nucleotide sequences could be relevant to enable the removal of ADP-ribosylation from specific substrates. Whether the hydrolases share similar residues that carry out key catalytic steps or whether their reaction mechanisms are fundamentally different is unknown. To define the hydrolytic mechanisms and the key residues responsible for the catalytic activity on ADP-ribosylated nucleic acids, structural resolution of ADP-ribosylated oligonucleotides in complex with the hydrolases will be necessary.

### Viral macrodomain-containing hydrolases

Macrodomains are also present in various viral families, including positive single-stranded (+)ssRNA viruses such as *Chikungunya virus* (CHIKV) and the *Corona viruses* (CoV). The function of these viral macrodomains is poorly understood, although it was shown that they efficiently reverse protein MARylation (126,134–137). Several studies found that the severe acute respiratory syndrome coronavirus (SARS-CoV) macrodomain Mac1 promotes viral replication *in vivo* and suppresses the interferon response, thus playing an important role in viral pathogenesis (136,138,139). In addition, PARP inhibitors enhance the replication of SARS-CoV and decrease interferon production during infection with a macrodomain mutant virus (140). Similarly, for CHIKV, the ADP-ribosylhydrolase activity appears essential for viral replication and virulence (141,142). Therefore, the viral macrodomains are thought to play essential roles in infectivity.

Uncapped mRNA in the cytoplasm can be recognized as a non-self RNA and trigger an innate immune response (143). Therefore, viruses deploy mechanisms to cap their mRNA, thereby mimicking the host cell cap and avoiding an innate immune response. The majority of (+)ssRNA viruses synthesize the canonical m7Gppp-type RNA cap, sometimes combined with 2′-*O*-me of the first nucleotide (cap-0 and cap-1, respectively), using their own set of capping enzymes (144,145). The canonical m7Gppp cap is essential for the initiation of translation and protects nascent RNA from premature degradation by 5′→3′ exonucleases (78). Although in general viral RNA capping is efficient, it also presents a sensitive step in the viral life cycle, reflected in the essential role of capping enzymes in viral replication. Capping of the viral RNA with a structurally different cap provides the host cell with an additional strategy to recognize and respond to viral RNA. The NAD^+^ cap blocks efficient translation of RNAs (80) and the structurally similar ADP-ribose cap could thus impair the translation of viral RNA. A recent study showed that PARP10 overexpression leads to decreased levels of processed viral non-structural proteins in cells (142), which could be due to impaired processing of the polyprotein as suggested, or due to lower protein translation from a potentially ADP-ribosylated viral RNA. Thus, viral macrodomains may have evolved to antagonize MARylation executed by PARPs that are responsive to interferon signalling. Future work will have to discern whether viral macrodomains also revert MARylation of vRNA as this might be a mark by the host to interfere with translation or transcription. Because the ADPr-cap is likely to interfere with m7Gppp-capping, a tight coordination of the viral de-MARylation and capping activities would be expected. Moreover, the ADPr-cap could serve as additional signal to stimulate the innate immune response. Key to unravel these possibilities is to follow up with studies in cells and evaluate whether and when RNA-capping by ADPr occurs. For this, the ability to selectively detect MARylation in cells is essential, which has been difficult and a limiting factor. Recent findings have provided information about newly developed tools that may help to measure this modification in cells, as discussed below.

## DETECTION METHODS

### *Detection of* in vitro *ADP-ribosylated substrates*

The ability of ARTs to transfer ADP-ribose from the co-factor NAD^+^ to their substrates can be exploited for the *in vitro* detection and identification of ADP-ribosylated proteins by employing modified NAD^+^ as substrate (1,146). Radiolabelled NAD^+^ offers two major benefits: first, the exchange of isotopes does not alter the chemical properties of NAD^+^ and, consequently, it should not influence enzymatic reactions; and second, the detection of radiolabelled substrates, including proteins and nucleic acids, is high sensitivity as demonstrated in multiple *in vitro* studies (55,68). Chemical NAD^+^ analogues with various functional groups attached to the NAD^+^ have been generated. In contrast to radiolabelled isotopes, the chemical and physical properties of modified NAD^+^ analogues are changed considerably compared to non-modified NAD^+^. Due to the release of nicotinamide during the reaction, NAD^+^ analogues commonly hold modifications at the adenosine group. Biotin-labelled NAD^+^ has been used to label proteins with different PARPs in several studies, for example on high-density protein microarrays (147–149) and as alternative to ^32^P-NAD^+^ in ADP-ribosylation reactions *in vitro* (150–153). From the published studies it appears that biotin-labelled NAD^+^ is well suited as cofactor.

### Detection of ADP-ribosylated substrates in cells

Methods to detect endogenously MARylated substrates are scarce. Early studies on the generation of MARylated protein specific antibodies yielded a rabbit serum with significant specificity for MARylated eEF2 (154). Moreover, using an ADPr-coupled carrier protein, a serum was obtained that recognized pertussis toxin modified G*α* as well as eEF2 (155). Then a rabbit serum (R-28) was developed specifically against ADP-ribosyl-arginine proteins, capable of distinctly identifying arginine-specific ADP-ribosylation products (156,157). Similarly, an anti-serum against ADP-ribosylated histones was generated and utilized for the *in vitro* characterization of ADP-ribosylation in murine T-cells as well as for the detection of endogenous ADP-ribosylation in rat skeletal muscle (158). Recently, a commercial PAR/MAR antibody has become available that enables studying MARylation (159). However, this antibody, as the name suggests, cannot distinguish between MARylation and PARylation and thus selective antibodies to detect MARylation are still lacking.

Catalytically inactive macrodomains form an alternative to antibodies and have been used as sensing tools to detect ADP-ribosylated proteins (160). The combination of affinity purification using ADPr binding macrodomains and subsequent mass spectrometry of the macrodomain-bound fraction enabled the discovery of both known and novel ADP-ribosylated proteins (161–163). The majority of these experiments were carried out using the Af1521 macrodomain present in *Archaeoglobus fulgidus*, which recognizes MARylated and PARylated substrates. Unknown at the time of the first mass spectrometry pull-downs, Af1521 is also an active hydrolase. Nevertheless, the pull-down experiments were successful due to their incubation at 4°C as low temperature seems to minimize its hydrolase activity (161,162). This macrodomain has been further developed to increase affinity (164). In contrast, the murine Parp14 macrodomains macro2/macro3 are catalytically inactive and interact preferentially with MAR (165). Therefore, the Parp14 macrodomains were optimized for co-immunoprecipitation and co-localization experiments selective for MARylated proteins (166). To analyse ADP-ribosylated proteins a fusion protein of a tandem Af1521 macrodomain (167), as well as the macrodomains2/3 from human PARP14 were generated (160).

**Figure 3.**
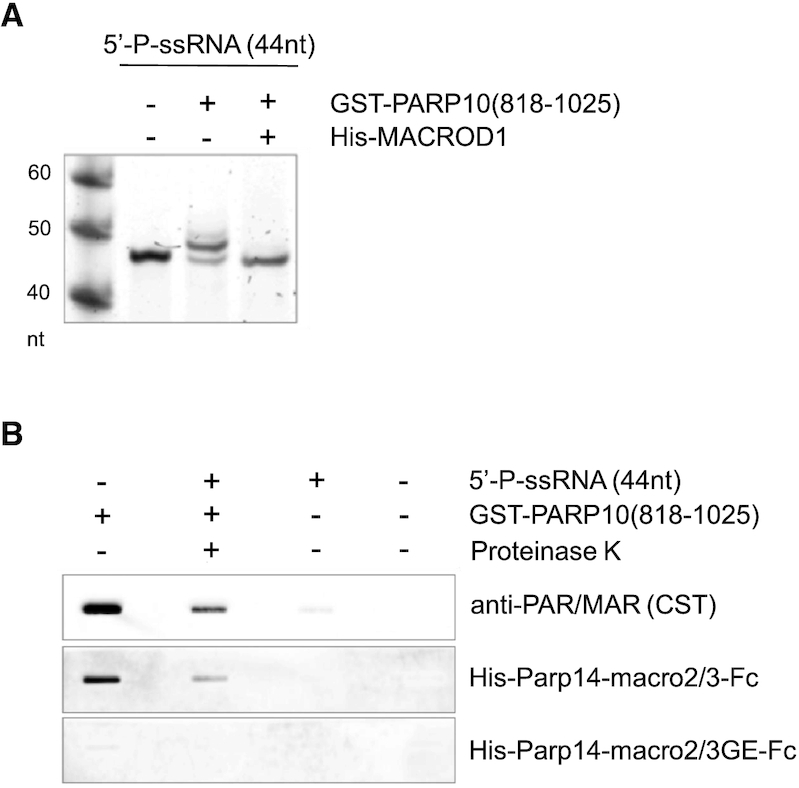
Detection of ADP-ribosylated RNA by immunostaining. (**A**) Mono-phosphorylated ssRNA (44 nucleotides) was incubated with PARP10(818–1025) and MACROD1 as indicated, followed by proteinase K treatment and RNA extraction. Samples were analysed on an urea–PAGE stained with SYBR-gold. (**B**) ADP-ribosylated oligonucleotides were blotted and incubated with the indicated detection reagents, followed by secondary antibodies and chemiluminescence detection. Ten ng ssRNA was loaded per slot. Controls: auto-modified PARP10(818–1025) without nucleic acid; buffer alone. Detection reagents used: anti-PAR/MAR, Cell Signalling Technology (CST) E6F6A; murine Parp14 macro2/macro3 wildtype or mutant (GE) fused to an Fc-tag for detection (166). PARP10(818–1025) corresponds to the catalytic domain of the enzyme (7). Data were generated in our laboratory (L. Weixler).

These reagents developed for detection of MARylated proteins have not yet been tested for their recognition of MARylated DNA or RNA. Preliminary experiments in our laboratory showed that some MAR reagents have the potential to detect ADP-ribosylated RNA. We modified ssRNA by incubation with the catalytic domain of PARP10 as reported before (68) (Figure 3A) and blotted the purified oligonucleotides to enable immunostaining. Different reagents that are available to read MARylation and PARylation recognize the modified RNA (Figure 3B). Considering the chemical structure of RNA and the ability of the macrodomain fold to bind RNA through a positively charged groove outside of the ADP-ribose binding site, appropriate controls should be applied to address specificity of MAR-binding reagents. The commercially available PAR/MAR antibody recognizes ADPr-RNA with high affinity. However, weak binding to non-modified RNA was also observed, limiting its use in studying RNA ADP-ribosylation in cells (Figure 3B). Compared to the PAR/MAR antibody, purified fusion proteins containing macro2/macro3 from murine Parp14 (166) and an Fc domain (Parp14-macro2/3-Fc) for detection, show a lower signal overall, but higher specificity for ADP-ribosylated RNA. No signal is observed with the Parp14-macro2/3-Fc mutant that is defined by glycine to glutamate mutations in the two macrodomains (166). These mutations block the access to the ADP-ribose binding site and prevent binding to ADPr. Despite the lower signal with Parp14-macro2/3-Fc in this slot blot analysis, it can potentially be employed to detect MARylated RNA as the mutant can be used to subtract any signal derived from binding independent of ADP-ribosylation. In addition to mere detection, this reagent can theoretically be used to precipitate specifically MARylated RNAs from a pool of cellular RNAs, to be identified by subsequent RNA-sequencing. This makes macro2/3 of Parp14 a potentially promising tool for studying RNA ADP-ribosylation to answer questions such as whether and when RNA ADP-ribosylation occurs in cells. We have not tested all available reagents, but have included the image as proof-of-principle that reagents developed to detect protein modification can be applied for the study of nucleic acids.

An alternative method for the detection of ADP-ribosylation of proteins, which does not rely on antibodies, utilizes clickable NAD^+^-analogues (168–170). After modification of targets, with for example 6-alkyne NAD^+^, functional tags for visualization or affinity tags for purification can be conjugated using click chemistry. Tagging occurs after the ADP-ribosylation reaction has taken place. In a similar approach, a subset of PARPs have been engineered to utilize only specific NAD^+^-analogues, enabling the detection of ADP-ribosylation incorporated exclusively by this specifically engineered enzyme (171–173). One drawback of these methods is the reliance on NAD^+^-analogues, which are not taken up by cells. ADP-ribosylation reactions have therefore been performed in cell lysates, which may enhance the occurrence of artefacts due to disruption of cellular structures. Recently, a clickable aminooxy probe (AO-alkyne) was developed, which together with an azide reporter enables monitoring of PARP activity also in live cells (174). This modification however relies on the ADP-ribosylated glutamate/aspartate side chain undergoing a 1′–2′ transfer of the ADP-ribose. Considering that the bond between ADP-ribose and nucleic acids differs from the bond between ADP-ribose and acidic amino acids (Figure 2), it needs to be determined whether this method might be adapted for the detection of nucleic acid modification. Considering the fact that the NAD^+^ analogues can be processed by the PARPs tested, it is plausible that also nucleic acids can be modified and studied using the mentioned methods, although procedures need to be optimized.

## FUTURE OUTLOOK

The modification of nucleic acids with ADP-ribose was first observed in organisms such as clams and cabbage butterflies, where DNA ADP-ribosylation appears to be part of the immune defence. Recent data highlight that also mammalian, intracellular ARTs can transfer ADP-ribose to nucleic acid substrates, both RNA and DNA, if carrying either a 5′- or a 3′-phosphate. Different transferases were reported to have different affinities towards a variety of substrates, partially depending on whether the full-length enzymes or truncations were used in the *in vitro* reactions (Tables 1 and 2). The different hydrolases reverse the modification regardless of oligonucleotide characteristics. Further research is required to address this apparent discrepancy: why are the hydrolases highly specific for either PAR or MAR, which is linked to certain amino acids, but promiscuous towards modified nucleic acids? Is this caused by simple steric hindrance of a bulky protein blocking access of the ADPr to the catalytic site?

Additional clarifications are required to understand the stimuli that activate the transferases to ADP-ribosylate nucleic acids, and to determine whether post-translational modifications or certain co-factors regulate the activities of different transferases. The activation mechanism of PARP1 has been studied in detail, whereas this information is lacking for the other family members. The fact that some truncated versions of PARPs, which solely harbour the catalytic domain, show increased activity towards nucleic acids when compared to the full-length proteins (68) suggests the presence of additional regulatory mechanisms. Moreover, it will be important to determine whether the activities of different PARPs towards protein and nucleic acid substrates can be regulated to favour one or the other class of substrates.

The demonstration of ADP-ribosylation of DNA and RNA *in vitro* has given rise to many questions concerning its relevance in cells. First of all, does it occur in mammalian cells at all? This question can now be addressed using the reagents described above; even though they were developed for protein ADP-ribosylation, they efficiently recognize *in vitro* modified oligonucleotides. In an initial approach, the reagents can be used to test whether ADP-ribosylation of DNA and RNA occurs under basal conditions in cells. Then it needs to be asked in which context this modification is introduced and removed in cells by which enzymes. Is DNA only modified when damaged, or can DNA ADP-ribosylation be part of other physiological processes, perhaps as a novel epigenetic regulator? Likewise, is RNA modified as a signal of potential errors introduced for example during transcription or splicing to halt subsequent translation, does it influence stability, or does it have other signalling functions? As a subset of PARPs is upregulated by interferons and interferes with viral replication, viral DNA and RNA are potential direct targets. This is particularly interesting to consider in light of the role of DNA ADP-ribosylation in pathogen defence and immunity in some of the discussed organisms. The modification of RNA with NAD^+^ is considered as a non-conventional cap (80), which does not support translation of the modified transcripts. ADP-ribose could serve similarly as non-conventional cap. When applied to a viral DNA or RNA for example, it could form a highly effective way of inhibiting viral replication. In this scenario, the macrodomains present in certain viruses could serve as antagonists to prevent ADP-ribosylation of viral RNA and to protect the RNA from being recognized by receptors of the innate immune system of the host cell.

**Figure 4.**
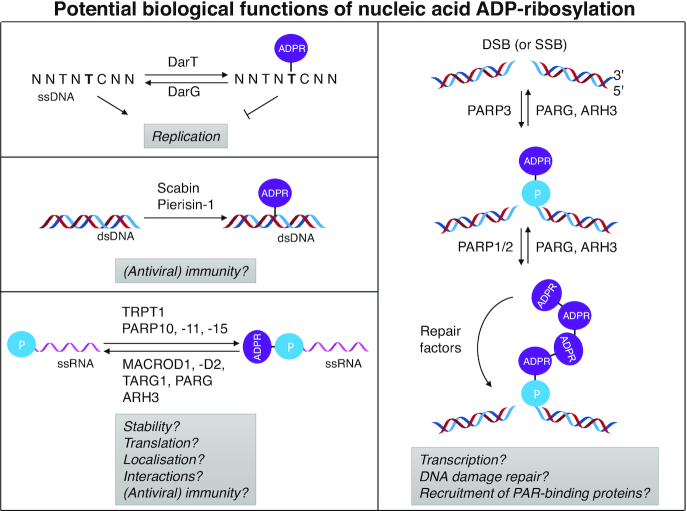
Potential *in vivo* functions of nucleic acid ADP-ribosylation. ADP-ribosyltransferases, hydrolases and their substrates are schematically displayed. Possible consequences of ADP-ribosylation are indicated with a question mark. The modification of double-stranded DNA by DarT leads to inhibition of replication, which is released by reversal of the modification by DarG. DNA modification in eukaryotes possibly play a role in the regulation of transcription, DNA damage repair and in the recruitment of PAR-binding proteins. Mono(ADP-ribosyl)ation by PARP3 can possibly be used as primer for PARP1/2 to generate poly(ADP-ribose). RNA modification may modify any RNA property, such as stability, translation, localization and interactome. This might not only apply to cellular RNAs, but also to foreign nucleic acids, such as viral RNA. These are the key questions that need to be addressed by future studies.

The stage is thus set for further explorations in the emerging field of nucleic acid ADP-ribosylation (Figure 4). Future studies should pursue two primary objectives: on the one hand, structural studies and biochemical *in vitro* studies are necessary to understand the regulatory mechanisms of the ADP-ribose metabolizing enzymes, to define their substrates and modes of substrate recognition; on the other hand, in cell and *in vivo* studies are required to clarify the presence of nucleic acid ADP-ribosylation, to verify the participation of the *in vitro* identified enzymes as well as to determine the consequences of ADP-ribosylation for nucleic acid substrates and downstream processes.

## DATA AVAILABILITY

All data is available in the manuscript; expression constructs are available on request.
